# Mobilization of hematopoietic and nonhematopoietic stem cell subpopulations in sepsis: a preliminary report

**DOI:** 10.1186/cc10615

**Published:** 2012-03-20

**Authors:** T Skirecki, U Zielińska-Borkowska, M Złotorowicz, M Złotorowicz, J Kawiak, G Hoser

**Affiliations:** 1 Medical Center of Postgraduate Education, Warsaw, Poland; 2 Polish Academy of Science, Warsaw, Poland

## Introduction

Sepsis and septic shock lead to the multiorgan damage by extensive release of inflammatory mediators. Regenerative mechanisms include such regimens as stem cells which differentiate towards specific tissues. Also, in the course of the systemic inflammation the disruption of various regulatory axes occurs, including chemokines (VEGF, HGF) and complement proteins (C5a,C3a). Among other functions these axes maintain stem cell circulation and recruitment [1]. The aim of the study was to evaluate circulating stem cells in the peripheral blood of septic patients.

## Methods

Blood samples were obtained from five patients with sepsis or septic shock on the second day after diagnosis. Blood from five healthy volunteers served as control. Samples were stained with the panel of antibodies against: CD45, lineage markers (Lin), CD34, CD133, VEGFR2 and isotypic controls. Cells were analyzed by flow cytometry and the total cell count per milliliter was calculated.

## Results

On the basis of cell surface phenotype the following stem cell subpopulations were distinguished: hematopoietic stem cells (HSCs) CD34^+^CD133^+^CD45^+^Lin^-^, endothelial progenitor cells (EPCs) CD34^+^CD133^+^VEGFR2^+^; and primitive nonhematopoietic stem cells. In the blood of septic patients we found: HSCs (5/5), median level 96/ml; EPCs (5/5), median 48/ml; and nonhematopoietic stem cells (4/5), median 48/ml. Whereas in the control group the results were as follow: HSCs (5/5), median level 644/ml; EPCs (5/5), median 70/ml; and nonhematopoietic stem cells (0/5). Two of five patients died of septic shock. A trend to lower number of HSCs in nonsurvivors was observed.

## Conclusion

Stem cells can be identified phenotypically in the blood of septic patients and healthy volunteers. However, the circulating primitive nonhematopoietic stem cells could not be detected under physiological conditions. Furthermore, we suggest that stem cells analysis may have serve as prognostic tool in the future.
